# Assessment of Response and Safety of Bulevirtide Treatment in Patients with Chronic Delta Virus Infection: The ARISTOTLE Pilot Observational Study

**DOI:** 10.3390/v17020251

**Published:** 2025-02-12

**Authors:** Luca Rinaldi, Mauro Viganò, Alessia Ciancio, Alfredo Caturano, Vincenzo Messina, Grazia Anna Niro, Nicolina Capoluongo, Alessandro Loglio, Letizia Marinaro, Aldo Marrone, Ernesto Claar, Maurizio Russello, Emanuela Ciracì, Umberto Vespasiani Gentilucci, Valeria Pace Palitti, Carlo Acierno, Clelia Cosentino, Andrea Mormone, Rosa Cotugno, Francesca Terracciani, Paolo Gallo, Maria Rita Cannavò, Valerio Rosato, Ferdinando Carlo Sasso, Chiara Petrucciello, Giulio Petronio Petronio, Giovanni Villone, Francesco Benanti, Giuseppe Cariti, Elisabetta Falbo, Marco Distefano, Rodolfo Sacco, Alessandro Perrella, Antonio Izzi

**Affiliations:** 1 Department of Medicine and Health Sciences “Vincenzo Tiberio”, Università degli Studi del Molise, 86100 Campobasso, Italy; luca.rinaldi@unimol.it (L.R.); c.petrucciello@studenti.unimol.it (C.P.); giulio.petroniopetronio@unimol.it (G.P.P.); giovanni.villone@unimol.it (G.V.); 2 Gastroenterology, Hepatology and Transplantation Division, ASST Papa Giovanni XXIII, 24127 Bergamo, Italy; mvigano@asst-pg23.it (M.V.); aloglio@asst-pg23.it (A.L.); 3 Department of Medical Sciences, University of Turin, 10124 Turin, Italy; alessia.ciancio@unito.it (A.C.); letizia.marinaro@gmail.com (L.M.); gcariti@hotmail.it (G.C.); 4 Department of Advanced Medical and Surgical Sciences, University of Campania Luigi Vanvitelli, 80138 Naples, Italy; aldo.marrone@unicampania.it (A.M.); clelia.cosentino@unicampania.it (C.C.); andrea.mormone@hotmail.it (A.M.); ferdinandocarlo.sasso@unicampania.it (F.C.S.); 5 Department of Human Sciences and Promotion of the Quality of Life, San Raffaele Roma Open University, 00166 Rome, Italy; 6 Infectious Diseases Unit, Sant’Anna e San Sebastiano Hospital, 81100 Caserta, Italy; sperimentazionicaserta@gmail.com; 7 Department of Gastroenterology, IRCCS Casa Sollievo della Sofferenza, 71013 San Giovanni Rotondo, Italy; g.niro@operapadrepio.it (G.A.N.); r.cotugno@operapadrepio.it (R.C.); 8 Department of Emergency Infectious Diseases and Infectious Diseases, Ospedali dei Colli, P.O.D. Cotugno, 80131 Naples, Italy; nicolina.copoluongo@ospedalideicolli.it (N.C.); alessandro.perrella@ospedalideicolli.it (A.P.); izziantonio@yahoo.it (A.I.); 9 Department of Medicine, Ospedale Evangelico Villa Betania, 80147 Naples, Italy; ernestoclaar@libero.it (E.C.); valeriorosato@gmail.com (V.R.); 10 Liver Unit, ARNAS Garibaldi Nesima, 95124 Catania, Italy; mrussello@arnasgaribaldi.it (M.R.); mariaritacannavo84@gmail.com (M.R.C.); 11 Internal Medicine Unit, Ostuni Hospital, 72017 Ostuni, Italy; emanuelaciraci@yahoo.it; 12 Clinical Medicine and Hepatology Unit, Fondazione Policlinico Universitario Campus Bio-Medico, 00128 Rome, Italyf.terracciani@unicampus.it (F.T.); paolo.gallo@policlinicocampus.it (P.G.); 13 Internal Medicine and Hepatology Unit, ASL Pescara, 65124 Pescara, Italy; vpacepalitti@gmail.com; 14 Department of Emergency Medicine, AOR San Carlo, 85100 Potenza, Italy; carlo894@gmail.com; 15 Unit of Infectious Diseases, Department of Clinical and Experimental Medicine, ARNAS Garibaldi Nesima Hospital, University of Catania, 95124 Catania, Italy; francesco.benanti61@gmail.com; 16 Travel and Migration Medicine Center, P.O. Lamezia Terme Hospital, 88046 Calabria, Italy; elisabetta.falbomd@gmail.com; 17 Hepatology Unit, ASP 8, 96100 Siracusa, Italy; erdis@tin.it; 18 Gastroenterology and Digestive Endoscopy Unit, Foggia University Hospital, 71122 Foggia, Italy; saccorodolfo@hotmail.com

**Keywords:** hepatitis delta virus, bulevirtide therapy, liver disease management, safety and efficacy, antiviral treatment outcomes

## Abstract

Introduction: Hepatitis D virus (HDV) infection remains a significant global health challenge due to its severity and high risk of progression to cirrhosis and hepatocellular carcinoma (HCC). Bulevirtide, a novel HDV entry inhibitor, has shown promise in managing chronic hepatitis D by blocking viral entry into hepatocytes. This study evaluated the efficacy and safety of bulevirtide in reducing HDV RNA levels and improving liver function in a real-life cohort of Italian patients with HDV infection. Methods: This multicenter prospective trial enrolled 108 consecutive patients with chronic HDV infection, from June 2023 to June 2024, who received 2 mg/day of bulevirtide in combination with a nucleoside/nucleotide analogue for hepatitis B virus (HBV) infection. Patients with any stage of liver fibrosis or compensated cirrhosis were included. Data collected included demographic and clinical characteristics, liver function tests, HDV RNA levels, and adverse events at baseline and 6 months. Results: The virological response was achieved in 54.6% of patients (*n* = 59), with 36 demonstrating undetectable HDV RNA levels. Among responders, ALT levels decreased significantly from 67.0 U/mL [IQR 44.0–116.3] to 31.5 U/mL [IQR 24.0–36.5, *p* = 0.001], and AST levels from 66.0 U/mL [IQR 46.5–91.0] to 32.5 U/mL [IQR 28.0–38.0, *p* = 0.021]. Median HDV RNA dropped from 29,800 IU/mL [IQR 3100–375,000] to 0 IU/mL [IQR 0–291, *p* < 0.001]. No significant predictors of response emerged. Mild adverse events, including pruritus (5.6%) and injection-site reactions (1.9%) and flu-like syndrome (0.9) were reported, with no treatment discontinuation. Conclusions: Bulevirtide effectively reduces HDV RNA levels and improves liver function with a favorable safety profile, offering a promising therapeutic option for chronic hepatitis D. Further large-scale studies are needed to confirm these findings and explore long-term outcomes.

## 1. Introduction

Delta hepatitis remains a significant global health issue, primarily due to its severity and progression [1]. Caused by the hepatitis D virus (HDV), which is the smallest human-infecting virus with a genome of approximately 1700 nucleotides forming a single-stranded RNA. This infection is transmitted parenterally and manifests as a particularly aggressive form of liver disease [1,2,3]. HDV is classified as a defective or incomplete [4] or satellite virus, requiring hepatitis B virus (HBV) surface antigen (HBsAg) and specifically the sodium taurocholate co-transporting polypeptide (NTCP) for infection and pathogenesis in human hepatocytes [5,6]. Global estimates indicate that 5% of HBV patients are co-infected with HDV, translating to around 12–14 million people, though prevalence varies with geography and socioeconomic factors [7,8,9]. The severity of HDV-related disease underscores the need for improved diagnostics and therapeutic approaches, with specific serological testing for anti-HDV antibodies and HDV RNA quantification by PCR being recommended [10,11,12,13].

Historically, Pegylated interferon-2a (PegIFN-2a) has been the cornerstone of HDV treatment, though its success rates are limited (10–20%) and it carries a risk of substantial side effects [14,15,16]. Bulevirtide (BLV)**,** an HDV entry inhibitor, presents a novel approach by competitively blocking NTCP, thereby preventing HDV entry into hepatocytes and mitigating viral replication and spreading though the liver [17]. Approved by the European Medicines Agency at a daily dose of 2 mg subcutaneously, BLV has shown promising results in terms of both efficacy and tolerability [18,19]. However, while clinical trials provide robust data under controlled settings, their findings may not fully capture the complexities of patient outcomes in real-world scenarios [20]. Factors such as comorbidities, variable adherence to treatment, and the heterogeneity of HDV patient populations, including differences in liver disease severity, geographic prevalence, and socioeconomic conditions, pose significant challenges to generalizing clinical trial results to broader, real-world contexts. These complexities underscore the need for observational studies that reflect the diversity and nuances of routine clinical practice [20]. To address these gaps, this study aims to evaluate the efficacy and safety of BLV in a cohort of Italian patients with HDV. By assessing changes in HDV RNA, liver enzyme levels, and other clinical parameters, it seeks to provide valuable insights into the long-term performance of BLV in a real-world setting, offering evidence that complements and extends the findings of controlled trials.

## 2. Materials and Methods

The Assessment of Response and Impact of Safety Treatment with bulevirtide in patients with chronic delta virus infection: an Observational Trial for Evaluating Long-term Efficacy (ARISTOTLE) study is multicenter, prospective, real-life pilot trial conducted at 13 hospitals and academic centers across Italy. The “pilot” designation reflects the study’s initial phase, with plans for future expansion of the cohort through the inclusion of additional patients from other centers. These future phases will also incorporate an extended follow-up period to evaluate long-term outcomes more comprehensively. From June 2023 to June 2024, all consecutive HDV-RNA-positive patients under the care of these centers were enrolled. A total of 126 patients initially met the eligibility criteria; however, one patient did not provide informed consent, and 18 patients did not complete the 6-month follow-up within the timeframe of this analysis, resulting in a final analysis of 108 patients.

Eligible patients included adults with chronic HDV infection who were serum HDV-RNA-positive and presented with any stage of liver fibrosis or compensated cirrhosis, as determined by clinical assessment, biochemical parameters, ultrasound findings, or FibroScan^®^ (Echosens, Paris, France). Liver stiffness, measured by transient elastography (FibroScan^®^), was categorized as indicating no or mild fibrosis within the range of 2.5 to 7 kPa [21], while values of 11 kPa or higher were considered indicative of advanced fibrosis [22]. The controlled attenuation parameter (CAP) thresholds used for stratifying liver steatosis were 248 dB/m for S1 (mild steatosis), 268 dB/m for S2 (moderate steatosis), and 280 dB/m for S3 (severe steatosis) [23]. Only patients meeting the eligibility criteria for BLV treatment under Italian ministerial guidelines were enrolled. These criteria included adult patients with chronic HDV infection, plasma (or serum) HDV-RNA positivity, and compensated liver disease. BLV was contraindicated in patients with decompensated liver disease (Child–Pugh score ≥B7) and in pregnant women. All enrolled patients were capable of completing a 6-month follow-up.

Patients were included regardless of HIV co-infection status or baseline hepatocellular carcinoma (HCC) diagnosis, with HCC screening conducted according to European Association for the Study of the Liver (EASL) guidelines [10]. Patients were excluded if they were under 18 years of age, pregnant, had co-infection with HCV, had decompensated cirrhosis, or had a history of liver transplantation.

Eligible patients received a daily dose of 2 mg of BLV, administered subcutaneously, either as monotherapy or combined with a nucleoside/nucleotide analogue to treat underlying hepatitis B virus (HBV) infection. Patients were treated with either tenofovir or entecavir according to standard treatment protocols for HBV infection. Tenofovir disoproxil fumarate (TDF) was administered orally at a dose of 245 mg once daily, while entecavir (ETV) was prescribed at a dose of 0.5 mg once daily for non-cirrhotic patients or 1 mg once daily for those with liver cirrhosis. This regimen followed criteria established by the European Medicines Agency Committee and adhered to current international guidelines [18,24].

Virological response to BLV was evaluated at the 6-month mark using real-time PCR to measure HDV-RNA levels. Two validated assays were used for quantification: the RoboGene^®^ HDV RNA Quantification Kit 2.0 (Roboscreen GmbH, Leipzig, Germany), with a detection limit of 6 IU/mL, and the RealStar^®^ HDV RT-PCR Kit 1.0 (altona Diagnostics GmbH, Hamburg, Germany), with a detection limit of 9.48 IU/mL. Patient safety was closely monitored through weekly structured interviews aimed at identifying and documenting any adverse effects throughout the study period. Laboratory tests assessing liver function, cytolysis indices, renal function, and hematopoietic function were documented in a shared database. If treatment discontinuation occurred due to adverse effects, the patient was withdrawn from the study. The study was conducted in accordance with the ethical principles outlined in the Declaration of Helsinki (1975) and its subsequent amendments. The study protocol received approval from the local ethics committee at the University of Molise (ID 41332, dated 11 September 2024). All participants provided written informed consent prior to enrollment.

### 2.1. Endpoints of Study

The primary endpoint of this study was a virological response to BLV at 6 months of treatment, assessed by real-time PCR. This endpoint was defined as achieving either undetectable serum HDV-RNA levels or a reduction in HDV-RNA by at least two logarithmic units from baseline, accompanied by normalization of transaminase levels [25]. The secondary endpoint focused on evaluating the safety profile of BLV, with adverse effects monitored through weekly structured interviews with patients.

### 2.2. Statistical Analysis

Continuous variables are presented as either mean and standard deviation (SD) or median and interquartile range (IQR), depending on the distribution of the data, which were assessed using the Shapiro–Wilk test. Categorical variables are reported as frequencies and percentages. The study population was stratified into responder and non-responder groups based on treatment response. To compare baseline characteristics between these groups, Student’s *t*-test or the Mann–Whitney U test was used for continuous variables, and the chi-square test (with Yates’ correction applied when necessary) was employed for categorical variables. A box plot analysis was performed to assess the variations in ALT, AST, HDV RNA, and vitamin D levels between the two groups at baseline and 6-month follow-up. Univariable and multivariable logistic regression analyses were conducted to evaluate the associations between baseline characteristics and treatment response, with odds ratios (ORs) and 95% confidence intervals (CIs) reported. A *p*-value < 0.05 was considered statistically significant. All statistical analyses were performed using RStudio^®^ 2024.09.1 software (RStudio, Boston, MA, USA).

## 3. Results

The baseline characteristics of the study population (*n* = 108) are shown in Table 1. The mean age of participants was 53.5 years (SD 12.2), with a majority of males (63.0%) and Italian nationality (62.0%). The median BMI was 24.3 kg/m^2^ (IQR 22.7–26.6), and 89.5% of the sample had cirrhosis. Liver stiffness, assessed through transient elastography, had a median of 12.8 kPa (IQR 9.8–17.6), while the mean CAP score was 214.7 dB/m (SD 57.6). The median HDV RNA level was 42,660 IU/mL, with an interquartile range (IQR) of 3205–367,420 IU/mL. Among patients, 8.3% had a history of HCC (3.7% active), and 35.2% had received previous interferon therapy. Treatment suppression of HBV DNA was achieved in 82.4% of the study sample.

**Figure 1:**
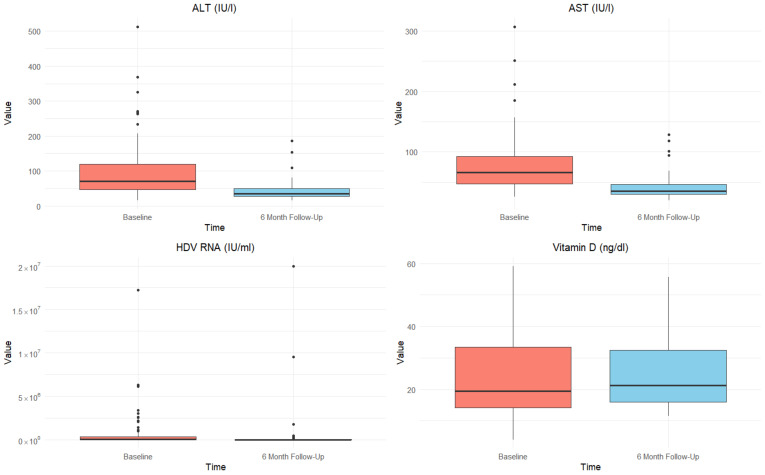
Box plot comparison of ALT, AST, HDV RNA, and vitamin D levels at baseline and 6-month follow-up.

Among patients treated with BLV 2 mg subcutaneously, 59 patients (54.6% of those enrolled) achieved the composite outcome. Of these, 36 patients demonstrated a complete virological response, while 23 showed a reduction in HDV-RNA levels by at least two logarithmic units along with normalization of transaminase levels. Table 2 provides a comparison of anthropometric and laboratory data at baseline and 6-month follow-up, stratified by treatment response. Table 2 and Figure 1 highlight the significant biochemical improvements observed among responders following BLV treatment. ALT levels demonstrated a significant reduction, decreasing from 67.0 U/mL (IQR 44.0–116.3) at baseline to 31.5 U/mL (IQR 24.0–36.5) at follow-up (*p* = 0.001). Similarly, AST levels declined significantly, from 66.0 U/mL (IQR 46.5–91.0) at baseline to 32.5 U/mL (IQR 28.0–38.0) at follow-up (*p* = 0.021), reflecting notable improvements in liver health. In contrast, non-responders exhibited only modest changes in these parameters, underlining the differential impact of the treatment between the two groups.

HDV RNA levels also showed profound reductions among responders, with median values decreasing from 29,800.0 IU/mL (IQR 3100.0–375,000.0) at baseline to undetectable levels in a subset by follow-up (median 0.0 IU/mL, IQR 0.0–291.0; *p* < 0.001), underscoring the virological efficacy of the therapy. While vitamin D levels increased in both groups, the changes did not reach statistical significance by the end of the follow-up period.

The box plots in Figure 1 illustrate these differences, highlighting significant reductions in ALT, AST, and HDV RNA levels among responders compared to non-responders, as well as an increase in vitamin D levels in both groups.

At multivariate logistic regression analysis, no clinical variables emerged to be associated with clinical response to BLV treatment (Table 3).

### Adverse Events and Safety Profile

A total of nine patients (8.4%) experienced adverse events associated with BLV treatment. The most frequently reported event was pruritus, occurring in six patients (5.6%). Injection-site reactions were reported in two patients (1.9%). One patient (0.9%) reported a flu-like syndrome accompanied by fatigue and anxiety. All adverse events were classified as mild in severity and did not necessitate drug discontinuation. No deaths occurred during the follow-up period.

## 4. Discussion

The main findings from this multicenter, prospective pilot study are as follows: BLV treatment effectively reduces HDV RNA levels in HDV-RNA-positive patients, with a substantial proportion achieving a virological response by the 6-month follow-up (55.7%) and 36 of them (33.3%) showing a complete virological response. No clinical variables were identified as being associated with treatment response. BLV was well-tolerated, with no adverse events leading to treatment discontinuation.

The reduction in or clearance of HDV RNA is a key finding, as it is closely linked to improved clinical outcomes, including enhanced liver function and a reduced risk of progression to cirrhosis and HCC [26]. In our study, responders also demonstrated significant reductions in ALT and AST levels, further supporting the efficacy of BLV in improving liver function. These decreases in liver enzymes not only reflect the antiviral effect of BLV, but have been also associated with reduced intrahepatic cytokines and inflammatory chemokine levels, further highlighting its role in mitigating liver inflammation [27], suggesting a positive impact on global liver health in HDV-infected patients.

Moreover, these findings are comparable to results from other clinical trials evaluating BLV [21,28,29]. Notably, even though there was no significant difference between responders and non-responders to treatment, our study observed a global improvement in both APRI score and FIB-4, two widely used non-invasive markers of liver fibrosis. This highlights BLV’s potential not just as a viral suppressant, but also as a therapeutic agent that may contribute to the long-term management of liver disease in HDV patients. The undetectable levels of HDV RNA in a subset of responders are especially noteworthy, as sustained virologic response (SVR) is a major endpoint in hepatitis therapies [30]. Data from a recent study on 96 weeks of BLV in patients with HDV-related cirrhosis demonstrate that virological and clinical responses improved over time, with few liver-related complications [31]. However, further long-term studies with larger populations are essential to confirm whether undetectable HDV RNA translates into improved outcomes, such as reduced cirrhosis progression or a lower incidence of HCC. These results, nonetheless, provide promising evidence that BLV may offer a pathway toward sustained remission in HDV infection. In addition to the reductions in HDV RNA and liver enzyme levels, our study observed an increase in HBsAg levels in both responders and non-responders. This finding is consistent with the results reported by Killer et al., who also noted an increase in HBsAg at 6 months, although it decreased at 12 months [32]. A possible explanation for this observation could be the competitive nature of HDV and HBV [33]. The reduction in HDV levels may alleviate the competition for cellular resources between the two viruses, thereby allowing HBV to increase its production of HBsAg. This interplay between HDV and HBV in the context of HBsAg production is complex and warrants further investigation.

In this study, no clinical variables were found to be significantly associated with the treatment response to BLV. This lack of association is somewhat unexpected, as prior research suggested that factors such as baseline HDV and HBV viral loads may influence antiviral treatment outcomes in chronic hepatitis D [34,35]. Moreover, vitamin D plays a crucial role in the immune response to viral infections [36]. It enhances the function of both the innate and adaptive immune systems, particularly through its effects on T-lymphocytes, which are essential for combating viral pathogens [37]. In viral infections, vitamin D deficiency has been associated with a weakened antiviral response, as it reduces the capacity of immune cells to effectively target and neutralize the virus [38].

In terms of safety, BLV demonstrated a favorable profile in this study. The majority of patients tolerated the drug well, with no major adverse events reported during the 6-month follow-up, which is consistent with previous studies highlighting the drug’s safety profile [16,21,28,31,32,33]. This is a significant advantage over conventional interferon-based therapies, which often come with a range of side effects, including flu-like symptoms, hematologic abnormalities, and gastrointestinal disturbances [39]. Peg-IFNα2a, a common treatment for HDV, is also associated with frequent side effects that may limit patient adherence [40]. In contrast, the BLV mechanism of action as an entry inhibitor of HDV could potentially offer a more targeted treatment with fewer side effects. Given its peptide-based nature and its specific action on HDV entry, BLV does not trigger the broad immune responses associated with interferons, making it a more tolerable option for patients [41]. This is particularly important in patients with advanced liver disease, who may already be suffering from various comorbidities.

### 4.1. Limitations

Despite the promising results, this study has several limitations that must be addressed. The sample size of 108 patients is relatively small, and further studies with larger cohorts are needed to confirm the generalizability of these findings. Additionally, the follow-up period was limited to 6 months, which, while adequate for assessing short-term responses, limits the ability to evaluate the long-term efficacy and safety of BLV. The impact of BLV on long-term liver outcomes, such as the progression to cirrhosis or the development of HCC, remains uncertain, and further long-term data are necessary. Another limitation of this study is the absence of direct histological evidence, such as liver biopsies, to corroborate improvements in liver health. While the study noted favorable changes in liver enzyme levels, these biochemical markers alone may not fully capture the extent of histological changes or the reversal of liver damage, potentially weakening claims about liver health improvements. Additionally, Fibroscan^®^ was not performed at the 6-month follow-up, and some assessments lacked CAP measurement due to software limitations. Furthermore, comprehensive HBV treatment histories were unavailable due to transitions between clinics and treatment complexity, but we included the regimen at enrollment and the type of nucleoside/nucleotide analogue used at BLV initiation. Finally, the potential for selection bias, particularly with regard to the patients’ nationality, should be considered. As highlighted, Italian nationality was associated with a lower likelihood of treatment response, and it is unclear whether this reflects a true biological difference or other confounding factors. Larger, more diverse studies are needed to determine whether this finding is replicable across different populations and to identify any other demographic or clinical factors that may influence treatment outcomes.

### 4.2. Future Directions

While the study provides important insights into the potential of BLV, further multicenter, larger-scale studies are necessary to confirm these findings and evaluate the long-term benefits and safety of BLV in diverse populations. Such studies should investigate the long-term impact of BLV on liver disease progression, including cirrhosis and HCC, and explore the potential of combining BLV with other antiviral therapies. Promising results already emerged from combination therapy with Peg-IFNα2a, which may enhance treatment outcomes and offer a more potent approach to managing HDV infection [42]. Additionally, personalized treatment strategies could help optimize the use of BLV in clinical practice by tailoring therapy to individual patient needs, taking into account both gene expression and polymorphisms [43,44]. We strongly believe that the future of HDV treatment has already begun due to the introduction of BLV in clinical practice, which provides a more targeted, effective, and tolerable approach compared to traditional therapies. Therefore, BLV represents a significant step forward in improving the liver health and the quality of life of such patients [45] living with this challenging and often fatal infection.

## 5. Conclusions

Bulevirtide appears to be a strong candidate towards an effective treatment for HDV infection. It effectively reduces HDV RNA levels and improves liver function, highlighting its therapeutic efficacy in managing both viral replication and liver inflammation.

## Figures and Tables

**Table 1:** Baseline characteristics of the study sample.

Variable	Baseline (*n* = 108)
Age, y, mean (SD)	53.5 (12.2)
Sex, *n* (%)	
M	68 (63.0)
F	40 (37.0)
Nationality, *n* (%)	
Italian	67 (62.0)
Romanian	22 (20.4)
Other	19 (17.6)
BMI, median [IQR]	24.3 [22.7–26.6]
Cirrhosis, *n* (%)	85 (89.5)
Stiffness (kPa), median [IQR]	12.8 [9.8–17.6]
Fibrosis stage, *n* (%)	
Absence	7 (6.5)
Mild	22 (20.4)
Advanced	63 (58.3)
Not performed	16 (14.8)
CAP (db/m), mean (SD)	214.7 (57.6)
Steatosis stage, *n* (%)	
S0	49 (45.4)
S1	7 (6.5)
S2	7 (6.5)
S3	5 (4.6)
Not performed/available	40 (37.0)
History of HCC, *n* (%)	9 (8.3)
Active	4 (3.7)
Previous interferon, *n* (%)	38 (35.2)
HDV RNA (UI/mL), median [IQR]	42,660 [3205–367,420]
HBV DNA not detected, *n* (%)	89 (82.4)
HBV treatment, *n* (%)	
Entecavir	52 (48.1)
Tenofovir	56 (51.9)
HCV treated, *n* (%)	9 (8.3)
HIV positive, *n* (%)	5 (4.6)

SD standard deviation; BMI body mass index; CAP controlled attenuated parameter; and HCC hepatocellular carcinoma.

**Table 2:** Comparison of baseline and 6-month follow-up anthropometric and laboratory data in the study sample.

Progression Time						
	Time 0 (Baseline)			Time 1 (=6 Months)		
Variable	Responder (*n* = 59)	Non-Responder (*n* = 49)	*p*	Responder (*n* = 59)	Non-Responder (*n* = 49)	*p*
PLT × 10^3^ (U/mL), median [IQR]	103.0 [78.0–153.0]	121.0 [80.0–183.0]	0.411	121.0 [80.0–183.0]	125.0 [95.0–170.0]	0.784
ALT (U/mL), median [IQR]	67.0 [44.0–116.3]	75.0 [47.8–125.3]	0.274	31.5 [24.0–36.5]	45.5 [34.0–64.0]	0.001
AST (U/mL), median [IQR]	66.0 [46.5–91.0]	66.0 [48.8–99.0]	0.772	32.5 [28.0–38.0]	43.0 [33.0–50.0]	0.021
ALP (U/mL), median [IQR]	88.0 [68.3–114.3]	91.0 [72.0–119.0]	0.858	82.0 [72.3–97.8]	87.0 [65.8–110.5]	0.996
GGT (U/mL), median [IQR]	59.0 [34.3–84.0]	63.0 [29.8–91.3]	0.939	37.0 [23.3–52.3]	40.5 [29.5–70.5]	0.226
Albumin (g/dL), median [IQR]	3.9 [3.6–4.2]	4.1 [3.8–4.4]	0.020	4.1 [3.9–4.4]	4.1 [3.8–4.4]	0.598
γ globulin (g/dL), median [IQR]	1.9 [1.5–2.2]	1.9 [1.4–2.4]	0.659	1.5 [1.2–1.8]	1.7 [1.2–1.9]	0.880
INR, median [IQR]	1.2 [1.1–1.4]	1.1 [1.0–1.2]	0.002	1.1 [1.1–1.2]	1.1 [1.0–1.2]	0.648
eGFR (mL/min/1.73 m^2^), median [IQR]	86.7 [69.3–98.5]	88.2 [78.0–98.6]	0.392	90.2 [74.3–100.7]	88.1 [75.0–100.6]	0.776
Vitamin D (ng/dL), median [IQR]	14.3 [11.0–21.6]	32.6 [19.1–39.7]	0.001	20.0 [14.9–29.2]	38.5 [16.4–47.0]	0.248
HBsAg (U/mL), median [IQR]	5722.0 [720.0–10,466.7]	4968.6 [887.0–9608.0]	0.813	8000.0 [1258.0–10,887.5]	9427.7 [4431.8–15,568.0]	0.188
APRI Score, median [IQR]	1.5 [1.0–2.4]	1.5 [0.8-2.5]	0.731	1.0 [0.5–2.4]	1.3 [0.8–1.3]	0.144
FIB-4, median [IQR]	3.7 [2.3–5.5]	2.9 [1.8–5.8]	0.205	2.7 [1.6–4.1]	2.7 [1.5–4.6]	0.895
HDV RNA (UI/mL), median [IQR]	29,800.0 [3100.0–375,000.0]	45,160.0 [3683.0–363,815.5]	0.615	0.0 * [0.0–291.0]	14,090.0 [1750.5–69,483.8]	<0.001

PLT platelets; IQR interquartile range; AST aspartate aminotransferase; ALT alanine aminotransferase; INR international normalized ratio; and eGFR estimated glomerular filtration rate. * HDV RNA levels of 0.0 indicate undetectable values, reflecting measurements below the detection limit of the assay.

**Table 3:** Univariable logistic regression model for treatment response.

	Univariable Analysis				Multivariable Analysis			
Parameter	OR	95% CI		*p*	OR	95% CI		*p*
Age	1.03	0.99	1.07	0.051	1.02	0.95	1.10	0.509
Sex	1.41	0.64	3.12	0.391				
M								
F								
Nationality								
Italian	1							
Romanian/Other	0.30	0.13	0.68	0.004	0.32	0.03	3.54	0.355
BMI	1.04	0.94	1.15	0.451				
Cirrhosis	0.79	0.32	1.95	0.606				
Stiffness	1.04	0.99	1.09	0.100				
History of HCC	0.78	0.20	3.11	0.726				
Previous interferon	0.60	0.15	2.47	0.479				
HBV DNA	1.00	0.99	1.01	0.638				
PLT	0.99	0.98	1.01	0.823				
ALT	0.99	0.99	1.01	0.312				
AST	1.00	0.99	1.01	0.846				
ALP	1.00	0.99	1.01	0.909				
GGT	0.99	0.98	1.01	0.606				
Albumin	0.32	0.12	0.84	0.020	1.36	0.15	12.28	0.935
γ globulin	0.80	0.31	2.03	0.634				
INR	1.57	0.86	2.68	0.245				
eGFR	0.99	0.97	1.02	0.357				
HBsAg	1.01	0.99	1.01	0.479				
APRI score	0.99	0.76	1.28	0.929				
FIB-4	1.02	0.90	1.17	0.717				
Vitamin D	0.92	0.87	0.98	0.013	0.93	0.88	1.02	0.182

BMI body mass index; HCC hepatocellular carcinoma; PLT platelets; ALT alanine aminotransferase; AST aspartate aminotransferase; ALP alkaline phenyl phosphatase; GGT gamma glutamil transpeptidasi; INR international normalized ratio; and eGFR estimated glomerular filtration rate.

## Data Availability

The data that support the findings of this study are available on reasonable request from the corresponding author.
